# Familial Clustering, Second Primary Cancers and Causes of Death in Penile, Vulvar and Vaginal Cancers

**DOI:** 10.1038/s41598-019-48399-4

**Published:** 2019-08-14

**Authors:** Luyao Zhang, Otto Hemminki, Tianhui Chen, Guoqiao Zheng, Asta Försti, Kristina Sundquist, Jan Sundquist, Kari Hemminki

**Affiliations:** 1 Division of Molecular Genetic Epidemiology, German Cancer Research Center (DKFZ), Im Neuenheimer Feld 580, D-69120 Heidelberg, Germany; 2 Faculty of Medicine, University of Heidelberg, Heidelberg, Germany; 3 Department of Urology, Helsinki University Hospital, Helsinki, Finland; 4 Cancer Gene Therapy Group, Faculty of Medicine, University of Helsinki, Helsinki, Finland; 5 Group of Molecular Epidemiology and Cancer Precision Prevention, Zhejiang Academy of Medical Sciences (ZJAMS), Hangzhou, China; 6 Center for Primary Health Care Research, Lund University, 205 02 Malmö, Sweden; 7 Department of Family Medicine and Community Health, Department of Population Health Science and Policy, Icahn School of Medicine at Mount Sinai, New York, USA; 8 Center for Community-based Healthcare Research and Education (CoHRE), Department of Functional Pathology, School of Medicine, Shimane University, Shimane, Japan

**Keywords:** Diseases, Oncology

## Abstract

Data on familial risks in penile and vulvar/vaginal cancers and in second primary cancers (SPCs) following these cancers are limited. We used the Swedish Family-Cancer Database from years 1958 through 2015 to identify 3641 penile and 8856 vulvar/vaginal cancers and to calculate relative risks (RRs) and 95% confidence intervals (CIs) for these cancers according to site-specific cancer in family members; additionally risk for SPCs was calculated. The familial RR for concordant (same) penile cancer was 3.22 (1.34–7.74), and it was 2.72 (1.69–4.39) for vulvar/vaginal cancer; RRs were increased for vulvar/vaginal cancer in families of anal cancer patients. RR for second penile cancer after penile cancers was 11.68 (7.95–17.18), while that for concordant vulvar/vaginal cancer was 9.03 (7.31–11.15). SPCs were diagnosed in 16.8% of penile cancer patients and in them 45.9% of deaths were caused by SPC (other than penile cancer). In vulvar/vaginal cancer patients with SPC, 36.4% of deaths were due to SPC. The results showed that these genital cancers might run in families and as SPCs are associated with human papilloma virus and smoking related cancers. Risk for these genital and anal SPCs are high and a follow-up plan should be agreed at diagnosis of these cancers.

## Introduction

Microbial infections are estimated to account for 16% of the global cancer burden, and of these 50% of female cancers are associated with human papillomavirus (HPV) infection while the share for male cancers is less than 5%^1,2^. The cancers for which evidence on the association with HPV infections is strongest include cervical, anogenital and a subset of upper aerodigestive tract cancers, particularly tonsillar and oropharyngeal cancers^3^. Penile cancers are very rare, with an incidence of 1.9/100,000 in Sweden^4^. HPV infections are associated probably with most cases and other risk factors include smoking, ultraviolet irradiation, chronic infections, warts and condylomas, phimosis and lack of circumcision^5^. The 5-year survival of penile cancer is around 70% in Europe and USA in the early 2000s^6,7^. The incidence of vulvar and vaginal cancers is 4.4/100,000, with more than half of tumors being located in the vulva, the rest in the vagina and in unspecified locations^4^. Risk factors for female genital cancers are essentially the same as for cervical cancer. HPV accounts for about half of the cases and other risk factors are immunosuppression, irradiation, other infections and smoking^8–10^. In a recent meta-analysis the average 5-year survival in HPV-positive vulvar cancer was 68% compared to 57% for HPV-negative vulvar cancer^10^. In contrast to many family studies of cervical cancer, data on familial clustering of penile, vaginal and vulvar cancers are very limited. In a study on HPV-related cancers published in 2008 we showed a risk for first-degree relatives for penile and vulvar cancers, and the latter was also associated with cervical cancer^9^. Similarly, studies on for second primary cancers (SPCs) following male and female genital cancers are rare while those following cervical cancer are more common, and generally shown an increased risk of HPV and smoking related sites and cancers in anatomic sites close to the cervix, probably as a result of treatment or more wide-spread infection^11–13^. SPCs following *in situ* cervical cancers showed the highest risks of anal and vulvar/vaginal cancers but also increased risks of upper aerodigestive tract, esophageal, lung and stomach cancers^11^.

Here we used the current update of the Swedish Family-Cancer Database, the largest family dataset in the world, to address familial risks, SPCs and mortality in penile, vulvar and vaginal cancers. For internal consistency we assessed familial risks bi-directionally (in reverse order), i.e., risk for penile cancer when family members were diagnosed with any cancer, and risk for any cancer when family members were diagnosed with penile cancer. For parent-offspring relationships the data are largely independent and confirmed bi-directional associations would be evidence for a true biological association. Similarly, risks for SPCs were analyzed bi-directionally, e.g., penile cancer either as first cancer or SPC after any other cancer. Mortality data were compared in penile, vulvar and vaginal cancers with and without SPCs, focusing on cancer causes of death, as it can be suspected that some SPCs are particularly fatal.

## Results

The Family-Cancer Database included 3641 penile and 8856 vulvar/vaginal cancers, diagnosed at median ages of 68 and 73 years, respectively (Table 1). Considering only the offspring generation (born after 1931) the respective case numbers were 1278 and 2233 with median ages at diagnosis of 58 and 59 years, respectively. The number of SPCs were 610 (16.8%) after penile and 1028 (11.6%) after vulvar/vagina cancers, diagnosed 7 and 5 years (medians) after the genital cancers.

**Table 1:** The number and age at diagnosis of patients with penile cancer and vulvar/vaginal cancer and number of patients with second primary cancer.

Primary cancer site	N (all/offspring)	Median age at diagnosis (years)
Penile cancer	3641/1276	68
Vulvar/vaginal cancer	8856/2233	73
**Second primary cancer**	**N** (**%**)	**Median follow**-**up time** (**years**)
After penile cancer	610^a^ (16.8)	7
After vulvar/vaginal cancer	1028^b^ (11.6)	5

^a^The number of second primary cancer in the offspring generation was 178, median follow-up time are 6 years.

^b^The number of second primary cancer in the offspring generation was 271, median follow-up time are 6 years.

In reference to RRs, we use the convention that we refer to risks only when they are significant, i.e., the 95%CIs do not overlap with the reference RR of 1.00. Accordingly, it is redundant to repeat 95%CIs in the text as these anyway are shown in the tables.

Table 2 shows familial risk for penile cancer in sons whose first-degree family members were diagnosed with any cancer, and in reverse order, the risk of cancer in the offspring generation when father or brother were diagnosed with penile cancers. At least 2 penile cancers (or a significant familial risk in any analyses) had to be recorded with any cancer in relatives (first column) for the site to be listed. The RR for penile cancer was increased to 3.22 when family members were diagnosed with the same (concordant) cancer (p-value < 0.05); familial cases were 2 brother pairs and 3 father-son pairs. The only other significant association of 3.30 was with bone cancer. In the reverse analysis, risk for any cancer in offspring when family members were diagnosed with penile cancer, the RRs for ovarian cancer (1.60) and thyroid gland tumors (1.79) were significant. As penile cancer is a rare cancer, no more but a single penile cancer was found in families of discordant cancers. Thus the number contributing families matches the number of ‘Familial cases’ given as the first column in Table 2.

**Table 2:** RRs for penile cancer when family members were diagnosed with any cancer, and RR for cancer when family members were diagnosed with penile cancer.

Cancer site family member	RR for penile cancer			RR for cancer		
	Familial cases	RR	95% CI	Familial cases	RR	95% CI
Upper aerodigestive tract	19	1.07	0.68–1.68	21	1.21	0.79–1.85
Esophagus	8	1.20	0.60–2.41	6	1.01	0.46–2.26
Stomach	33	1.08	0.76–1.53	16	1.24	0.76–2.03
Small intestine	3	0.82	0.26–2.54	2	0.55	0.14–2.21
Colorectum	100	1.11	0.91–1.37	62	0.84	0.65–1.07
Anus	1	0.56	0.08–4.01	1	0.46	0.06–3.25
Liver	27	1.29	0.88–1.89	9	0.67	0.35–1.28
Pancreas	27	1.23	0.84–1.80	16	1.05	0.65–1.72
Nose	0	—	—	1	0.88	0.12–6.24
Lung	71	1.23	0.97–1.56	55	1.00	0.76–1.30
Breast (female and male)	98	0.95	0.78–1.17	139	0.97	0.82–1.15
Cervix	20	1.42	0.91–2.20	20	1.33	0.86–2.06
Endometrium	23	1.00	0.66–1.51	21	0.94	0.61–1.44
Uterus, unspecified	3	1.14	0.37–3.55	3	1.16	0.37–3.60
Ovary	22	1.20	0.78–1.82	30	**1.60**	**1.12–2.29**
Vulvar/vaginal	6	1.62	0.72–3.61	4	1.51	0.56–4.02
Prostate	124	0.97	0.81–1.17	109	0.82	0.68–0.99
Penile	5	**3.22**	**1.34–7.74**			
Kidney	17	0.72	0.44–1.15	14	0.73	0.43–1.23
Urinary bladder	37	0.97	0.70–1.35	33	1.10	0.78–1.55
Melanoma	29	1.07	0.74–1.55	51	1.04	0.79–1.36
Skin	38	1.07	0.78–1.48	22	0.85	0.56–1.30
Nervous system	27	1.15	0.78–1.68	43	1.21	0.90–1.64
Thyroid gland	7	1.09	0.52–2.28	17	**1.79**	**1.12–2.89**
Endocrine glands	15	1.09	0.65–1.81	13	0.74	0.43–1.28
Bone	4	**3.30**	**1.24–8.81**	2	0.76	0.19–3.03
Connective tissue	7	1.37	0.65–2.87	7	1.14	0.55–2.40
Non-Hodgkin lymphoma	22	0.88	0.58–1.34	33	1.21	0.86–1.71
Hodgkin lymphoma	3	0.84	0.27–2.60	3	0.47	0.15–1.46
Myeloma	9	0.76	0.39–1.46	8	0.92	0.46–1.84
Leukemia	20	0.87	0.56–1.35	27	1.07	0.74–1.57
CUP^a^	23	0.89	0.59–1.34	9	0.48	0.25–0.92
All (including penile)	628	**1.12**	**1.00–1.25**	815	0.97	0.91–1.04

CUP^a^ = cancer of unknown primary. RR = relative risk, 95% CI = 95% confidence interval, bold font indicates that the lower limit of 95% CI does not include 1.00.

Table 3 shows familial risk for vulvar/vaginal cancer in daughters whose first-degree family members were diagnosed with any cancer, and in reverse order, the risk of cancer in the offspring generation when mother or sister were diagnosed with vulvar/vaginal cancers. The combined numbers of familial cases were about two times higher than in Table 2. The RR for vulvar/vaginal cancer was increased to 2.72 when family members were diagnosed with the same cancer (p-value < 0.0001); familial cases were 6 sister pairs and 11 mother-daughter pairs. The other significant associations were with anal (2.38), liver (1.34), nasal (2.68) and lung (1.32) cancers. In the reverse analysis, RRs for anal (2.13), unspecified uterine (1.98) and bladder (1.29) cancers were increased. Similar to penile cancer in Table 2, the number of contributing families is equal to the number of ‘Familial cases’ given as the first column.

**Table 3:** RRs for vulvar/vaginal cancer when family members were diagnosed with any cancer, and RR for cancer when family members were diagnosed with vulvar/vaginal cancer.

Cancer site family member	RR for vulvar/vaginal cancer			RR for cancer		
	Familial cases	RR^b^	95% CI^c^	Familial cases	RR	95% CI
Upper aerodigestive tract	32	1.03	0.73–1.47	49	1.18	0.89–1.56
Salivary glands	4	1.23	0.46–3.28	5	1.08	0.45–2.61
Esophagus	9	0.80	0.41–1.53	13	0.90	0.52–1.56
Stomach	63	1.23	0.96–1.58	29	0.94	0.65–1.36
Small intestine	5	0.82	0.34–1.98	7	0.81	0.38–1.69
Colorectum	150	0.98	0.83–1.15	175	0.98	0.85–1.14
Anus	7	**2.38**	**1.14–5.01**	11	**2.13**	**1.18–3.85**
Liver	48	**1.34**	**1.01–1.78**	22	0.68	0.45–1.03
Pancreas	35	0.94	0.67–1.31	38	1.04	0.75–1.42
Nose	6	**2.68**	**1.20–5.97**	4	1.48	0.56–3.96
Lung	130	**1.32**	**1.10–1.57**	151	1.13	0.96–1.32
Breast (female and male)	166	0.96	0.82–1.13	312	0.94	0.84–1.05
Cervix	24	0.99	0.66–1.48	24	0.71	0.48–1.07
Endometrium	48	1.25	0.94–1.66	62	1.16	0.90–1.48
Uterus, unspecified	2	0.47	0.12–1.89	12	**1.98**	**1.12–3.49**
Ovary	27	0.88	0.60–1.29	39	0.89	0.65–1.22
Vulvar/vaginal	17	**2.72**	**1.69–4.39**			
Prostate	191	0.90	0.77–1.04	273	0.84	0.75–0.95
Testis	3	0.70	0.23–2.17	18	0.92	0.58–1.46
Penile	4	1.51	0.56–4.02	6	1.62	0.72–3.61
Kidney	41	1.03	0.76–1.41	48	1.05	0.79–1.40
Urinary bladder	72	1.12	0.89–1.42	94	**1.29**	**1.05–1.58**
Melanoma	57	1.24	0.95–1.61	90	0.80	0.65–0.98
Skin	59	0.98	0.76–1.27	62	0.99	0.77–1.27
Eye	2	0.50	0.12–1.99	5	0.82	0.34–1.96
Nervous system	48	1.23	0.93–1.64	72	0.90	0.72–1.14
Thyroid gland	10	0.91	0.49–1.70	14	0.67	0.39–1.13
Endocrine glands	25	1.08	0.73–1.60	37	0.92	0.66–1.27
Bone	4	1.94	0.73–5.17	2	0.36	0.09–1.43
Connective tissue	11	1.27	0.70–2.30	18	1.30	0.82–2.07
Non-Hodgkin lymphoma	38	0.90	0.66–1.25	65	1.02	0.80–1.30
Hodgkin lymphoma	6	0.98	0.44–2.19	11	0.81	0.45–1.46
Myeloma	19	0.95	0.60–1.49	18	0.86	0.54–1.37
Leukemia	39	1.00	0.73–1.37	44	0.77	0.57–1.03
CUP^a^	42	0.95	0.70–1.28	32	0.71	0.50–1.00
All (including vulvar/vaginal)	1027	1.05	0.96–1.14	1880	0.95	0.91–1.00

CUP^a^ = cancer of unknown primary. RR^b^ = relative risk. 95% CI^c^ = 95% confidence interval, bold font indicates that the lower limit of 95% CI does not include 1.00.

Risks for SPCs are shown in Table 4 after penile cancer (i.e., in survivors of penile cancer) and for penile cancer as the SPC (i.e., in survivors of any primary cancer). Only risks of penile (11.68) and anal (3.97) cancers were increased. In the reversed analysis, risk for penile cancer was not increased after any other cancer.

**Table 4:** RRs of second primary cancers (SPCs) in survivors of penile cancer and other cancers.

Cancer site	After penile cancer			After other cancers		
	SPCs	RR^b^	95% CI^c^	SPCs	RR	95% CI
Upper aerodigestive tract	19	1.12	0.72–1.76	14	0.64	0.38–1.08
Salivary gland	0	—	—	0	—	—
Esophagus	8	1.01	0.51–2.02	1	0.37	0.05–2.60
Stomach	24	0.76	0.52–1.14	10	0.51	0.28–0.95
Small intestine	3	1.10	0.35–3.40	1	0.33	0.05–2.34
Colorectum	70	0.90	0.71–1.13	38	0.35	0.26–0.49
Anus	3	**3.97**	**1.28–12.31**	4	2.19	0.82–5.84
Liver	14	0.83	0.49–1.39	2	0.29	0.07–1.18
Pancreas	19	1.08	0.69–1.70	0	—	—
Nose	0	—	—	0	—	—
Lung	69	1.23	0.97–1.56	4	0.18	0.07–0.49
Breast	2	1.96	0.49–7.85	1	0.01	0.001–0.04
Prostate	175	0.85	0.73–0.99	123	0.82	0.69–0.99
Testis	2	2.06	0.52–8.25	6	1.64	0.74–3.65
Penile	26	**11.68**	**7.95–17.18**			
Kidney	12	0.66	0.37–1.16	3	0.14	0.05–0.45
Urinary bladder	47	1.03	0.78–1.37	28	0.57	0.39–0.82
Melanoma	12	0.72	0.41–1.28	19	0.51	0.32–0.79
Skin	46	1.11	0.83–1.49	36	0.70	0.21–0.98
Eye	0	—	—	1	0.32	0.04–2.24
Nervous system	9	0.88	0.46–1.69	4	0.20	0.08–0.54
Thyroid gland	0	—	—	3	0.32	0.10–0.99
Endocrine glands	3	0.71	0.23–2.21	3	0.14	0.05–0.43
Bone	1	1.77	0.25–12.59	1	0.71	0.10–5.04
Connective tissue	5	1.44	0.60–3.46	4	0.71	0.27–1.88
Non-Hodgkin lymphoma	14	0.75	0.44–1.26	13	0.61	0.36–1.06
Hodgkin lymphoma	2	1.07	0.27–4.26	2	0.62	0.16–2.49
Myeloma	1	0.11	0.01–0.75	2	0.26	0.06–1.03
Leukemia	12	0.65	0.37–1.14	5	0.27	0.11–0.65
CUP^a^	12	0.67	0.38–1.18	8	0.85	0.43–1.71
All (excluding penile)	584	**1.08**	**1.00–1.18**	336	0.42	0.38–0.47

CUP^a^ = cancer of unknown primary. RR^b^ = relative risk. 95% CI^c^ = 95% confidence interval, bold font indicates that the lower limit of 95% CI does not include 1.00.

In Table 5 SPCs after vulvar/vaginal cancers were analyzed, with significant risks for upper aerodigestive tract (2.26), anal (10.31), lung (1.81), cervical (2.38), vulvar/vaginal (9.03) and connective tissue cancers (3.64). In the reverse order, risks for vulvar/vaginal cancers were increased after anal (2.31), cervical (4.20), endometrial (1.52) and unspecified uterine cancers (2.13).

**Table 5:** RRs of second primary cancers (SPCs) in survivors of vulvar/vaginal cancer and other cancers.

Cancer site	After vulvar/vaginal cancer			After other cancers		
	SPCs	RR^b^	95% CI^c^	SPCs	RR	95% CI
Upper aerodigestive tract	26	2.26	1.54–3.32	14	0.33	0.20–0.56
Salivary gland	1	0.58	0.08–4.12	4	0.72	0.27–1.91
Esophagus	7	1.23	0.59–2.58	0	—	—
Stomach	28	0.85	0.59–1.23	15	0.38	0.23–0.63
Small intestine	7	1.80	0.86–3.78	0	—	—
Colorectum	119	1.02	0.85–1.22	136	0.65	0.55–0.77
Anus	26	**10.31**	**7.01–15.16**	8	**2.31**	**1.16–4.63**
Liver	28	0.87	0.60–1.26	5	0.37	0.15–0.88
Pancreas	26	0.91	0.62–1.33	2	0.17	0.04–0.68
Nose	1	0.80	0.11–5.71	0	—	—
Lung	81	**1.81**	**1.45–2.25**	9	0.22	0.11–0.41
Breast	173	0.92	0.79–1.06	226	0.73	0.64–0.83
Cervix	34	**2.38**	**1.70–3.33**	170	**4.20**	**3.61–4.89**
Endometrium	37	0.82	0.59–1.13	136	**1.52**	**1.29–1.80**
Uterus, unspecified	3	0.67	0.22–2.07	14	**2.13**	**1.26–3.60**
Ovary	24	0.73	0.49–1.08	51	1.31	0.99–1.72
Vulvar/vaginal	87	**9.03**	**7.31–11.15**			
Kidney	28	1.34	0.92–1.94	11	0.27	0.15–0.49
Urinary bladder	45	1.82	1.36–2.44	38	0.40	0.29–0.54
Melanoma	23	0.99	0.66–1.49	34	0.50	0.35–0.69
Skin	59	1.19	0.92–1.54	66	0.66	0.52–0.85
Eye	4	2.15	0.80–5.72	2	0.33	0.08–1.30
Nervous system	17	0.88	0.55–1.41	17	0.45	0.28–0.73
Thyroid gland	8	1.17	0.58–2.34	6	0.34	0.15–0.75
Endocrine glands	12	0.83	0.47–1.46	29	0.71	0.50–1.03
Bone	1	0.83	0.47–1.46	2	0.76	0.19–3.04
Connective tissue	17	**3.64**	**2.26–5.86**	6	0.56	0.25–1.24
Non-Hodgkin lymphoma	26	1.04	0.71–1.53	16	0.40	0.24–0.65
Hodgkin lymphoma	4	1.62	0.61–4.33	5	0.83	0.35–2.00
Myeloma	7	0.55	0.26–1.15	6	0.39	0.18–0.88
Leukemia	24	1.02	0.68–1.52	13	0.36	0.21–0.63
CUP^a^	45	1.20	0.89–1.60	11	0.59	0.33–1.07
All (excluding vulvar/vaginal)	941	**1.28**	**1.20–1.37**	1052	0.75	0.70–0.80

CUP^a^ = cancer of unknown primary. RR^b^ = relative risk. 95% CI^c^ = 95% confidence interval, bold font indicates that the lower limit of 95% CI does not include 1.00.

In Table 6 data are shown for concordant SPCs after primary penile (i.e., second penile cancer after primary penile cancer) and vaginal/vulvar cancer according to the follow-up time. The highest risk of second penile cancer was 16.23 at > 5 years of follow-up. For second vulvar/vaginal cancer the highest RR of 9.16 was reached at follow-up of >5 years but the RRs were quite uniform throughout.

**Table 6:** RRs of second primary penile and vulvar/vaginal cancers by follow-up time.

<=1 yr^a^			1–5 yr			>5 yr		
**After penile cancer**								
Cases	RR^b^	95% CI^c^	Cases	RR	95% CI	Cases	RR	95% CI
5	10.38	4.32–24.95	2	3.51	0.88–14.03	19	16.23	10.33–25.49
**After vulvar/vaginal cancer**								
Cases	RR	95% CI	Cases	RR	95% CI	Cases	RR	95% CI
22	8.90	5.85–13.50	22	8.92	5.87–13.56	43	9.16	6.79–12.35

yr^a^ = year. RR^b^ = relative risk. 95%CI^c^ = 95% confidence interval, bold font indicates that the lower limit of 95% CI does not include 1.00.

Causes of death in genital cancer patients are reported in Table 7. For penile cancer in patients without SPC, 65.7% of deaths were due to non-neoplastic causes and 28.4% were due to penile cancer; 5.9% were caused by cancers which were reported in death notifications but not in the cancer registry. For vulvar/vaginal cancers, 43.5% of deaths were caused by vulvar/vaginal cancer and 45.3% were due to non-neoplastic causes. For male patients with SPC, 45.9% of deaths were caused by SPC (other than penile cancer), higher order primaries accounted for 4.8% of deaths and non-neoplastic causes 32.9%. For female patients, SPCs (other than vulvar/vaginal cancers) caused 36.4% of deaths, higher order primaries 3.1% and non-neoplastic cause 32.4%.

**Table 7:** Cause of death in penile cancer and vaginal/vulvar cancer with or without second primary cancer.

Cause of death		Without second primary cancer	
		Penile cancer (N%)	Vulvar/vaginal cancer (N%)
Penile cancer		625 (28.4)	—
Vulvar/vaginal cancer		—	2754 (43.5)
Other cancers		131 (5.9)	707 (11.2)
Non-neoplastic		1447(65.7)	2869 (45.3)
All		2203 (100.0)	6330 (100.0)
**Cause of death**		**With second primary cancer**	
		**Penile cancer (N%)**	**Vulvar/vaginal cancer (N%)**
Penile cancer^a^		45 (9.4)	—
Vulvar/vaginal cancer^a^		—	145 (17.2)
SPC^b^	non-penile cancer	219 (45.9)	—
SPC^b^	non-vulvar/vaginal cancer	—	307 (36.4)
Higher order primary		23 (4.8)	26 (3.1)
Other cancers		33 (6.9)	92 (10.9)
Non-neoplastic		157 (32.9)	273 (32.4)
All		477 (100.0)	843 (100.0)

Penile cancer^a^ = including first primary cancer and second primary cancer.

Vulvar/vaginal cancer^a^ = including first primary cancer and second primary cancer.

SPC^b^ = second primary cancer.

Causes of death in penile cancer patients are listed according to the type of SPC (Supplementary Table 1). Prostate, lung and colorectal cancers as SPC caused the highest numbers of deaths due to SPC. The most fatal SPCs were pancreatic (15 of 17 total deaths) and esophageal cancer (7 of 8 total deaths). Similar data for vulvar/vaginal cancer patients are shown in Supplementary Table 2. Breast, colorectal and lung cancers as SPC caused the highest numbers of deaths due to SPC. The most fatal SPC was lung cancer (60 of 74 total deaths) but also pancreatic and esophageal cancers claimed a high toll.

## Discussion

The present results confirmed the previous findings on concordant familial risk for penile cancer and vulvar/vaginal cancers with larger case numbers^9^. These risks were at the equal magnitude, 3.22, for penile cancer (p < 0.05) and 2.72 for vulvar/vaginal cancer (p < 0.0001) but with three times more familial cancers for the latter which also showed more than 2 times higher overall case numbers. Penile cancers were associated in single analyses with bone, ovarian and thyroid cancers but as solitary results there was no support against chance findings. Vulvar/vaginal cancers were associated bi-directionally with anal cancer families and uni-directionally with liver, nasal, lung, unspecified uterine and bladder cancer families. Both penile cancer and vulvar/vaginal cancer showed the highest risks as respective SPCs, together with high risk of anal cancer which for women was bi-directional. Cervical cancer was also bi-directionally associated with vulvar/vaginal cancer as SPC. Upper aerodigestive tract, lung and connective tissue cancers were increased as SPCs after vulvar/vaginal cancer, and the risk for vulvar/vaginal cancer was increased as SPC after endometrial and unspecified uterine cancers. Finally, we showed that SPCs had implications for mortality. SPCs were diagnosed in 16.8% of penile cancer patients and in these patients 45.9% of deaths were caused by SPC (other than penile cancer) compared to 32.9% of deaths due to non-neoplastic causes, which were the main cause of deaths (65.7%) in patients without SPC. In vulvar/vaginal cancer patients the effect of SPC was not as marked as in men but even in women with SPC, SPC was the main cause of death, accounting for 36.4% of all deaths.

Limitations of the study include small case numbers and hence large 95%CIs, even in spite of this being the largest study so far conducted on these cancers relating to familial and SPC risks. Another limitation is the lack of possibilities for external validation as much of the previous literature originates from the earlier versions of the Swedish database. However, as HPV is an important etiological factor for both penile and vulvar/vaginal cancers, the equal findings for the two cancers with consistent bi-directional associations should make a strong case for biological interpretations. In regard to concordant SPCs it is an acknowledged problem to distinguish recurrent primaries from independent primaries, particularly when an infective agent (HPV) is likely to be an overwhelming oncogenic driver. However the present results showing the higher risks for second penile and vulvar/vaginal cancers in longest follow-up time support the notion of independent primaries.

How can we consolidate the findings? Inherited genetic factors are likely to contribute to the associations, and for example in cervical cancer certain human leukocyte antigen (HLA) alleles show strong risk^14,15^. However, we need to consider also the contribution by other non-genetic risk factors, such as HPV-infection and smoking. First we note that the study involved many comparisons (over 30 different cancers were included) and chance findings are likely. In the same token we need to point out that many case numbers were small and for these the power of detection was small^16^. Formal correction for the number of comparisons, such as Bonferroni correction, is not useful for the present kind of data where most risk estimates were underpowered^17^. Thus we rely on internal consistency between bi-directional results and between familial and SPC results, and on biological plausibility. The possibility to invoke external consistency is meager because the previous literature largely originates from earlier versions of the present data sources. Risks for concordant genital cancers (familial and SPC) suggest the HPV-infection is the likely driver together with inherited genetic factors of these associations with a possible contribution with life-style factors such as smoking. Such mechanisms would also be plausible explanations for the observed association with cervical, lung and upper aerodigestive tract cancers. The increased risk of vulvar/vaginal cancers as SPC after cervical and unspecified uterine cancers may be caused by side effects of radiation after treatment of primary cancers, as suggested in previous studies^11–13^. Immunosuppression is often suggested to be a contributing mechanism for infection-related familial or personal risks^8^. Based on cancers arising in immunosuppressed patients, squamous cell skin cancer and non-Hodgkin lymphoma are considered hallmark cancers of dysfunctional immune system^18,19^. It is noteworthy that we found no evidence on increased associations of penile or vulvar/vaginal cancers with these cancers, in spite of reasonable case numbers; thus immune suppression appears not to be important in the present context.

How can we rationalize these results? HPV infections are usually sexually transmitted while for a familial risk this mode of transmission would be limited to relatively rare events, such as sexual abuse situations. Transmission from an infected mother during pregnancy or early childhood may take place but overall such non-sexual transmissions are thought to be rare^20^. We assume that shared life-style is the main explanation to the observed familial risks. In our previous studies we reported, for example, that husbands of women with cervical cancer had an increase in tobacco and HPV related cancers^21^. Risks for cervical and other female genital cancers, together with upper aerodigestive tract, anal, liver, pancreatic, lung, kidney and urinary bladder cancers were increased in women who had children with different men in Sweden^22^. Similarly for men who had children with different partners, increasing risks were shown for upper aerodigestive tract, lung, urinary bladder and esophageal cancers^23^. Risks for cervical and lung cancers were increased also in Swedish divorced women^24^.

The results on mortality overall support the moderate survival in the present genital cancers, consistent with non-neoplastic causes being the main cause of death, particularly for penile cancers. With improving survival, SPCs are becoming increasingly common and we showed that they had a negative influence on mortality. Cancers of known to be fatal as first primary cancers, such as pancreatic, esophageal and lung cancers, were also fatal as SPC.

In summary, we provide evidence that penile cancers and vulvar/vaginal cancers might be associated in families and as SPCs with HPV and smoking related cancers. Individual counseling about life-style related cancers may not be successful but overall health campaigns may bring some benefit. However, risk for concordant penile and vulvar/vaginal cancer and anal SPCs are high and a follow-up plan should be agreed at diagnosis of male and female genital cancer patients.

## Methods

### Database and cancer ascertainment

We used the update of The Swedish Family-Cancer Database which covered cancer data from 1958 through 2015 and family links over a century^25^. This Database includes 16 million individuals, covering the offspring generations born after 1931 and their biological parents (the parental generation) in some 4 million nuclear families. Siblings could be identified only in the offspring generation which reached a maximal age of 83 years in 2015.

Coverage of cytologically or histologically verified incident cancers is considered to over 90% complete on account of compulsory nationwide registration by clinicians and pathologists^26,27^. Cancer diagnoses were based on the 7^th^ or later revisions of the International Classification of Diseases (ICD). In ICD-10, the code for penile cancer was C60, for vulvar cancer it was C51 and for vaginal cancer it was C52. The Swedish Cancer Registry requests notifications for SPCs which are thought to be independent primary cancers, considering e. g., anatomic location, time since the first cancer and histology. For histologically identical cancers at the same organ site the independence cannot be clinically judged, and in these cases some SPCs may be recurrences, causing overestimation, However, it is not known how many true SPCs are not notified because the clinician considers them to be recurrences, leading to underestimation.

### Statistical analysis

Relative risks (RRs) were used to measure cancer risks for penile or vulvar/vaginal cancer in the offspring generation according to occurrence of any cancers in their first-degree family members (parents or siblings). Risks were assessed for offspring cancer depending on family history. Thus the RR for e.g., familial penile cancer was calculated for offspring cases of penile cancer when their family members were diagnosed with penile cancer considering their accumulated person-years at risk and compared to offspring penile cancers in families where no penile cancers were diagnosed. In the reverse analysis, RR was calculated for cancer in offspring when family members were diagnosed with genital cancer. These two types of analyses were partially independent, particularly for discordant cancers, and positive results in both analyses provided strong support for a true association^28^. Follow-up was started for each offspring at birth, immigration or January 1^st^, 1958, whichever came latest. Follow-up was terminated on diagnosis of first cancer, death, emigration, or the closing date of the study, which was December 31^st^, 2015. Poisson regression modeling was employed to estimate RRs and corresponding 95% confidence intervals (CI). For concordant familial RRs we calculated additionally the p-values to help inference about the significance of the results. Potential confounders, including sex, age group (5-year bands), period (5-year bands), socioeconomic status (blue-collar worker, white-collar worker, farmer, private, professional, or other/unspecified), residential area (large cities, South Sweden, North Sweden, or unspecified) were added to the model as covariates^28^.

RRs for SPCs were obtained by comparing incidence rates for cancer X as SPC in penile or vulvar/vaginal cancer patients with rates for cancer X as first cancer in the general population. In the reverse analyses, RRs were calculated for penile or vulvar/vagina cancer as the SPC following any cancer. Follow-up was started at diagnosis of the relevant genital cancer and terminated at diagnosis of SPC, emigration, death or December 31, 2015, whichever occurred first. Identical follow-up times were applied when genital cancer was considered as SPC. Sex, age group, calendar-period, socio-economic status and residential areas were treated as potential confounders and were adjusted for in the regression model^28^.

All cancer related deaths were stratified into penile or vulvar/vagina cancer, SPC, ‘other cancer’ and non-neoplastic cause of death. ‘Other cancer’ includes cases diagnosed at the issue of death certificates, referred to ‘death certificate notifications’^29^. These notifications are not used by the Swedish Cancer Registry to complement cancer data^26,29^. We have found that the notifications often included multiple cancers and cancer of unknown primary (CUP). In our previous studies we have used these as information on metastases^30^. If the death certificate notification matched the organ site of the reported primary cancer it was classified to that site but in most cases such an assignment could not be made and the classification was to ‘other neoplasia’^28^.

All statistical analyses were done with the SAS version 9.4.

### Ethical statement

The study was approved by the Ethical Committee of Lund University (Reg. No. 2012/795), Sweden, and the study was conducted in accordance with the approved guidelines not requesting informed consent^31^. The study is national register-based study on anonymous personal data.

## Supplementary information

Supplementary Dataset 1

## References

[1] Zur Hausen H (2009). The search for infectious causes of human cancers: where and why. Virology.

[2] Oh JK, Weiderpass E (2014). Infection and cancer: global distribution and burden of diseases. Annals of global health.

[3] IARC. Biological agents. Volume 100 B (2012). A review of human carcinogens. IARC monographs on the evaluation of carcinogenic risks to humans.

[4] CentreforEpidemiology. *Cancer incidence in Sweden 2012*. (The National Board of Health and Welfare, 2013).

[5] Eble, J., Sauter, G., Epstein, J. & Sesterhenn, I. In *WHO Classification of Tumors*, *Pathology & Genetics* 359 (IARC Press, Lyon, 2003).

[6] Verhoeven RH (2013). Population-based survival of penile cancer patients in Europe and the United States of America: no improvement since 1990. Eur J Cancer.

[7] Arya M (2013). Long-term trends in incidence, survival and mortality of primary penile cancer in England. Cancer Causes Control.

[8] Tavassoli, F. & Devilee, P. In *WHO Classification of Tumours* 432 (IARC Press, Lyon, 2003).

[9] Hussain SK, Sundquist J, Hemminki K (2008). Familial clustering of cancer at human papillomavirus-associated sites according to the Swedish Family-Cancer Database. Int J Cancer.

[10] Rasmussen CL, Sand FL, Hoffmann Frederiksen M, Kaae Andersen K, Kjaer SK (2018). Does HPV status influence survival after vulvar cancer?. Int J Cancer.

[11] Hemminki K, Dong C, Vaittinen P (2000). Second primary cancer after *in situ* and invasive cervical cancer. Epidemiology.

[12] Arnold M (2014). Second primary cancers in survivors of cervical cancer in The Netherlands: Implications for prevention and surveillance. Radiotherapy and oncology: journal of the European Society for Therapeutic Radiology and Oncology.

[13] Teng CJ (2015). Secondary Primary Malignancy Risk in Patients With Cervical Cancer in Taiwan: A Nationwide Population-Based Study. Medicine.

[14] Leo PJ (2018). Correction: Defining the genetic susceptibility to cervical neoplasia-A genome-wide association study. PLoS Genet.

[15] Bao X (2018). HLA and KIR Associations of Cervical Neoplasia. The Journal of infectious diseases.

[16] Hemminki, K., Sundquist, J. & Brandt, A. Do discordant cancers share familial susceptibility? *Eur J Cancer* **48**, 1200–1207, doi:S0959-8049(11)00728-3 [pii], 10.1016/j.ejca.2011.09.017 (2012). 10.1016/j.ejca.2011.09.017 22033325

[17] Nakagawa S (2004). A farewell to Bonferroni: the problems of low statistical power and publication bias. Behavioral Ecology.

[18] Birkeland S (1995). Cancer risk after renal transplantation in the Nordic countries, 1964–1986. Int J Cancer.

[19] Wimmer CD (2007). The janus face of immunosuppression - de novo malignancy after renal transplantation: the experience of the Transplantation Center Munich. Kidney Int.

[20] Syrjanen S (2010). Current concepts on human papillomavirus infections in children. Apmis.

[21] Hemminki K, Dong C (2000). Cancer in husbands of cervical cancer patients. Epidemiology.

[22] Li X, Hemminki K (2002). Cancer risks in women who had children with different partners from the Swedish Family-Cancer Database. Eur J Cancer Prev.

[23] Li X, Hemminki K (2003). Cancer risks in men who had children with different partners from the Swedish Family-Cancer Database. Eur J Cancer Prev.

[24] Hemminki K, Li X (2003). Life-style and cancer: effect of widowhood and divorce. Cancer Epidemiol Biomarkers Prev.

[25] Hemminki K, Ji J, Brandt A, Mousavi SM, Sundquist J (2010). The Swedish Family-Cancer Database 2009: Prospects for histology-specific and immigrant studies. Int J Cancer.

[26] Ji J, Sundquist K, Sundquist J, Hemminki K (2012). Comparability of cancer identification among Death Registry, Cancer Registry and Hospital Discharge Registry. Int J Cancer.

[27] Zhang L (2019). Second cancers and causes of death in patients with testicular cancer in Sweden. PLoS One.

[28] Zheng G, Chattopadhyay S, Forsti A, Sundquist K, Hemminki K (2018). Familial risks of second primary cancers and mortality in ovarian cancer patients. Clinical epidemiology.

[29] Pukkala E (2018). Nordic Cancer Registries - an overview of their procedures and data comparability. Acta Oncol.

[30] Riihimaki M, Hemminki A, Sundquist J, Hemminki K (2016). Patterns of metastasis in colon and rectal cancer. Scientific reports.

[31] Zheng G (2019). Second primary cancers in patients with acute lymphoblastic, chronic lymhocytic and hairy cell leukemia. Br J Dermatol.

