# Supplementary material for: Familial Clustering, Second Primary Cancers and Causes of Death in Penile, Vulvar and Vaginal Cancers
Source: Sci Rep. 2019 Aug 14;9:11804. doi: 10.1038/s41598-019-48399-4 (PMC6694134; doi:10.1038/s41598-019-48399-4)
Supplement: Supplementary file 1 — Supplementary Dataset 1 [file 41598_2019_48399_MOESM1_ESM.doc]

**SUPPLEMENTARY DATA**

**FAMILIAL CLUSTERING, SECOND PRIMARY CANCERS AND CAUSES OF DEATH IN**

**PENILE, VULVAR AND VAGINAL CANCERS**

**Luyao Zhang ^1,2^, Otto Hemminki ^3,4^, Tianhui Chen ^5^, Guoqiao Zheng ^1,2^, Asta Försti ^1,6^, Kristina Sundquist ^6,7,8^ Jan Sundquist ^6,7,8^ and Kari Hemminki ^1,5^**

**Supplementary** **Table 1** Causes of death in patients diagnosed with second primary cancer after male genital cancer

| **Second primary cancer** | **Total number of death patients** | **Cause of death (N%)** | | | | |
| --- | --- | --- | --- | --- | --- | --- |
|  |  | **MGC^1^** | **SPC^2^** | **HOPC^3^** | **OC^4^** | **Non-neoplastic** |
| Upper aerodigestive tract | 12 | 0 | 5 (41.7) | 0 | 1 (8.3) | 6 (50.0) |
| Esophagus | 8 | 0 | 7 (87.5) | 1 (12.5) | 0 | 0 |
| Stomach | 23 | 1 (4.3) | 17 (73.9) | 1 (4.3) | 1 (4.3) | 3 (13.0) |
| Small intestine | 3 | 0 | 0 | 0 | 1 (33.3) | 2 (66.7) |
| Colorectum | 58 | 3 (5.2) | 31 (53.4) | 7 (12.1) | 1 (1.7) | 16 (27.6) |
| Anus | 2 | 0 | 2 (100.0) | 0 | 0 | 0 |
| Liver | 14 | 2 (14.3) | 5 (35.7) | 0 | 2 (14.3) | 5 (35.7) |
| Pancreas | 17 | 2 (11.8) | 15 (88.2) | 0 | 0 | 0 |
| Lung | 60 | 2 (3.3) | 41 (68.3) | 2 (3.3) | 4 (6.7) | 11 (18.3) |
| Breast | 1 | 0 | 0 | 0 | 0 | 1 (100.0) |
| Prostate | 129 | 14 (10.9) | 54 (41.9) | 6 (4.7) | 4 (3.1) | 51 (39.5) |
| Other male genital | 18 | 6 (33.3) | 0 | 1 (5.6) | 1 (5.6) | 10 (55.6) |
| Kidney | 9 | 1 (11.1) | 5 (55.6) | 0 | 0 | 3 (33.3) |
| Urinary bladder | 37 | 3 (8.1) | 14 (37.8) | 2 (5.4) | 4 (10.8) | 14 (37.8) |
| Melanoma | 6 | 0 | 1 (16.7) | 0 | 2 (33.3) | 3 (50.0) |
| Skin, squamous cell | 28 | 5 (17.9) | 1 (3.6) | 2 (7.1) | 3 (10.7) | 17 (60.7) |
| Nervous system | 7 | 0 | 4 (57.1) | 0 | 3 (42.9) | 0 |
| Endocrine glands | 3 | 0 | 0 | 0 | 0 | 3 (100.0) |
| Bone | 1 | 0 | 0 | 0 | 1 (100.0) | 0 |
| Connective tissue | 5 | 0 | 2 (40.0) | 0 | 0 | 3 (60.0) |
| Non-Hodgkin lymphoma | 11 | 1 (9.1) | 4 (36.4) | 0 | 2 (18.2) | 4 (36.4) |
| Hodgkin lymphoma | 2 | 0 | 1 (50.0) | 0 | 0 | 1 (50.0) |
| Myeloma | 1 | 0 | 1 (100.0) | 0 | 0 | 0 |
| Leukemia | 10 | 1 (10.0) | 6 (60.0) | 0 | 0 | 3 (30.0) |
| Acute myeloid leukemia | 3 | 0 | 3 (100.0) | 0 | 0 | 0 |
| Acute lymphoid leukemia | 1 | 0 | 1 (100.0) | 0 | 0 | 0 |
| Chronic lymphoid leukemia | 3 | 1 (33.3) | 0 | 0 | 0 | 2 (66.7) |
| Others | 3 | 0 | 2 (66.7) | 0 | 0 | 1 (33.3) |
| CUP^5^ | 12 | 4 (33.3) | 3 (25.0) | 1 (8.3) | 3 (25.0) | 1 (8.3) |
| All | 477 | 45 (9.4) | 219 (45.9) | 23 (4.8) | 33 (6.9) | 157 (32.9) |

MGC^1=^male genital cancer.

SPC^2^=second primary cancer. HOPC^3^=higher (3th, 4th) order primary cancer.

OC^4^=other cancer (excluding second primary cancer and higher order primary cancer and male genital cancer).

CUP^5^=cancer of unknown primary.

**Supplementary** **Table 2** Causes of death in patients diagnosed with second primary cancer after female genital cancer

| **Second primary cancer** | **Total number of death patients** | **Cause of death (N%)** | | | | |
| --- | --- | --- | --- | --- | --- | --- |
|  |  | **FGC^1^** | **SPC^2^** | **HOPC^3^** | **OC^4^** | **Non-neoplastic** |
| Upper aerodigestive tract | 21 | 4 (19.0) | 6 (28.6) | 2 (9.5) | 1 (4.8) | 8 (38.1) |
| Esophagus | 7 | 1 (14.3) | 5 (71.4) | 0 | 1 (14.3) | 0 |
| Stomach | 27 | 2 (7.4) | 15 (55.6) | 0 | 2 (7.4) | 8 (29.6) |
| Small intestine | 6 | 1 (16.7) | 2 (33.3) | 0 | 1 (16.7) | 2 (33.3) |
| Colorectum | 100 | 15 (15.0) | 51 (51.0) | 0 | 3 (30.0) | 31 (31.0) |
| Anus | 19 | 2 (10.5) | 4 (21.1) | 1 (5.3) | 5 (26.3) | 7 (36.8) |
| Liver | 26 | 1 (3.8) | 16 (61.5) | 1 (3.8) | 1 (3.8) | 7 (26.9) |
| Pancreas | 26 | 1 (3.8) | 20 (76.9) | 0 | 2 (7.7) | 3 (11.5) |
| Nose | 1 | 1 (100.0) | 0 | 0 | 0 | 0 |
| Lung | 74 | 1 (1.4) | 60 (81.1) | 2 (2.7) | 4 (5.4) | 7 (9.5) |
| Breast | 129 | 19 (14.7) | 38 (29.5) | 8 (6.2) | 10 (7.8) | 54 (41.9) |
| Cervix | 29 | 5 (17.2) | 11 (37.9) | 1 (3.4) | 2 (6.9) | 10 (34.5) |
| Endometrium | 28 | 7 (25.0) | 6 (21.4) | 1 (3.6) | 6 (21.4) | 8 (28.6) |
| Uterus, unspecified | 2 | 0 | 0 | 1 (50.0) | 0 | 1 (50.0) |
| Ovary | 22 | 3 (13.6) | 11 (50.0) | 3 (13.6) | 0 | 5 (22.7) |
| Other female genital | 61 | 35 (57.4) | 0 | 0 | 4 (6.6) | 22 (36.1) |
| Kidney | 23 | 4 (17.4) | 10 (43.5) | 0 | 1 (4.3) | 8 (34.8) |
| Urinary bladder | 42 | 10 (23.8) | 14 (33.3) | 1 (2.4) | 3 (7.1) | 14 (33.3) |
| Melanoma | 12 | 2 (16.7) | 1 (8.3) | 0 | 0 | 9 (75.0) |
| Skin, squamous cell | 46 | 14 (30.4) | 0 | 1 (2.2) | 4 (8.7) | 27 (58.7) |
| Eye | 4 | 0 | 2 (50.0) | 0 | 1 (25.0) | 1 (25.0) |
| Nervous system | 15 | 1 (6.7) | 4 (26.7) | 0 | 3 (20.0) | 7 (46.7) |
| Thyroid gland | 5 | 0 | 2 (40.0) | 0 | 0 | 3 (60.0) |
| Endocrine glands | 7 | 2 (28.6) | 1 (14.3) | 0 | 0 | 4 (57.1) |
| Bone | 1 | 0 | 0 | 0 | 1 (100.0) | 0 |
| Connective tissue | 17 | 1 (5.9) | 2 (11.8) | 1 (5.9) | 8 (47.1) | 5 (29.4) |
| Non-Hodgkin lymphoma | 22 | 2 (9.1) | 2 (9.1) | 2 (9.1) | 10 (45.5) | 6 (27.3) |
| Hodgkin lymphoma | 3 | 1 (33.3) | 0 | 0 | 0 | 2 (66.7) |
| Myeloma | 7 | 0 | 6 (85.7) | 0 | 0 | 1 (14.3) |
| Leukemia | 21 | 2 (9.5) | 14 (66.7) | 1 (4.8) | 1 (4.8) | 3 (14.3) |
| Acute myeloid leukemia | 9 | 2 (22.2) | 7 (77.8) | 0 | 0 | 0 |
| Chronic myeloid leukemia | 1 | 0 | 1 (100.0) | 0 | 0 | 0 |
| Acute lymphoid leukemia | 1 | 0 | 1 (100.0) | 0 | 0 | 0 |
| Chronic lymphoid leukemia | 8 | 0 | 4 (50.0) | 1 (12.5) | 1 (12.5) | 2 (25.0) |
| Others | 2 | 0 | 1 (50.0) | 0 | 0 | 1 (50.0) |
| CUP^5^ | 40 | 8 (20.0) | 4 (10.0) | 0 | 18 (45.0) | 10 (25.0) |
| All | 843 | 145 (17.2) | 307 (36.4) | 26 (3.1) | 92 (10.9) | 273 (32.4) |

FGC^1=^female genital cancer.

SPC^2^=second primary cancer. HOPC^3^=higher (3th, 4th) order primary cancer.

OC^4^=other cancer (excluding second primary cancer and higher order primary cancer and female genital cancer).

CUP^5^=cancer of unknown primary.
